# Cationic porphyrins with large side arm substituents as resonance light scattering ratiometric probes for specific recognition of nucleic acid G-quadruplexes

**DOI:** 10.1093/nar/gkz064

**Published:** 2019-02-04

**Authors:** Li-Ming Zhang, Yun-Xi Cui, Li-Na Zhu, Jun-Qing Chu, De-Ming Kong

**Affiliations:** 1 Department of Chemistry, School of Science, Tianjin University, Tianjin 300072, China; 2 Tianjin Key Laboratory of Biosensing and Molecular Recognition, College of Chemistry, Nankai University, Tianjin 300071, China

## Abstract

Specific G-quadruplex-probing is crucial for both biological sciences and biosensing applications. Most reported probes are focused on fluorescent or colorimetric recognition of G-quadruplexes. Herein, for the first time, we reported a new specific G-quadruplex-probing technique—resonance light scattering (RLS)-based ratiometric recognition. To achieve the RLS probing of G-quadruplexes in the important physiological pH range of 7.4-6.0, four water soluble cationic porphyrin derivatives, including an unreported octa-cationic porphyrin, with large side arm substituents were synthesized and developed as RLS probes. These RLS probes were demonstrated to work well for ratiometric recognition of G-quadruplexes with high specificity against single- and double-stranded DNAs, including long double-stranded ones. The working mechanism was speculated to be based on the RLS signal changes caused by porphyrin protonation that was promoted by the end-stacking of porphyrins on G-quadruplexes. This work adds an important member in G-quadruplex probe family, thus providing a useful tool for studies on G-quadruplex-related events concerning G-quadruplex formation, destruction and changes in size, shape and aggregation. As a proof-of-concept example of applications, the RLS probes were demonstrated to work well for label-free and sequence-specific sensing of microRNA. This work also provides a simple and useful way for the preparation of cationic porphyrins with high charges.

## INTRODUCTION

G-quadruplexes are noncanonical nucleic acid secondary structure formed by guanine (G)-rich DNAs or RNAs. Since genomic sequences with G-quadruplex-forming potential are widely found in many important regions of human genome and increasing evidences reveal that G-quadruplex formation in these regions is closely related with some crucial biological functions (1–8), G-quadruplexes represent promising targets for development of new anticancer drugs (9–16). Corresponding research is pushed to a new climax from 2013 since substantive evidence was provided by Balasubramanian *et al.* for the G-quadruplex formation in human cells (17).

Besides biological and pharmaceutical interests, G-quadruplexes have also been widely used in other fields. A well-known one is biosensing application. To date, many G-quadruplex-based colorimetric, fluorescent, electrochemical and chemiluminescent biosensors have been reported (18–24). No matter for biological sciences or biosensing applications, searching for an excellent G-quadruplex probe with high G-quadruplex recognition specificity against other nucleic acid structures is a necessary prerequisite. Through the efforts of many researchers, a large number of G-quadruplex probes showing excellent G-quadruplex recognition performance have been reported (25–34). However, most of these probes are focused on the fluorescent recognition of G-quadruplexes, and the one that can achieve G-quadruplex-probing via resonance light scattering (RLS) signals has never been reported.

RLS is an unorthodox spectral analysis technology proposed by Pasternack *et al.* (35,36), Due to the characteristics of high sensitivity, good selectivity and convenience in operation, this technique got rapid development in recent years and has been widely used in the analysis of various targets, including nucleic acids, proteins, polysaccharides and other biological macromolecules (37). Luo and Li *et al.* have innovatively applied RLS technique in specific detection of parallel G-quadruplexes (38). Since the generation of RLS signal was relied on the formation of G-wire superstructures by parallel G-quadruplexes but not on the specific recognition by G-quadruplex probes, the reported method has limited applications, it could only work for parallel G-quadruplexes.

**Scheme 1.**
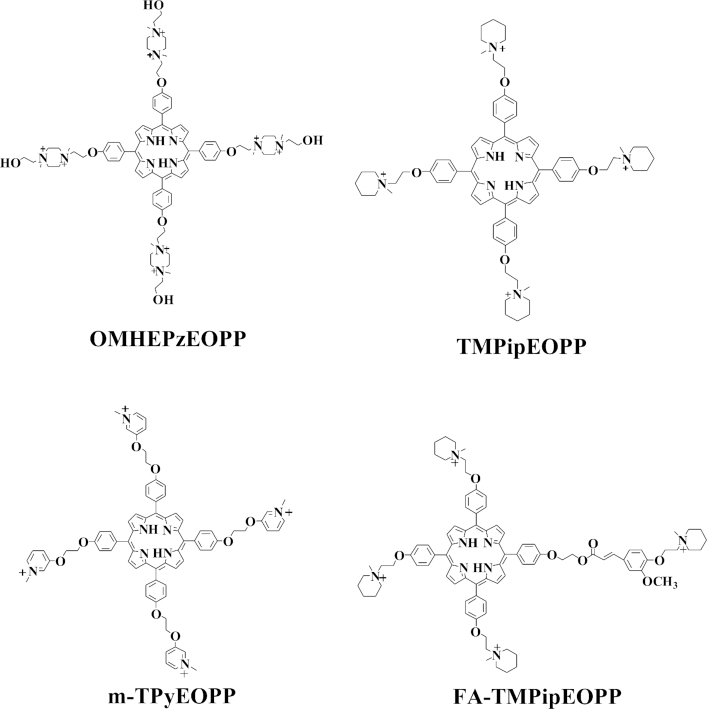
The chemical structures of the four porphyrin derivatives used in this work.

In our previous works (39–43), we have demonstrated that cationic porphyrin derivatives with large side arm substituents can be used as outstanding probes for colorimetric and fluorescent recognition of G-quadruplexes with high specificity. Herein, we found that these water soluble cationic porphyrin derivatives can also be developed as RLS probes for the ratiometric probing of G-quadruplexes. In most cases, the G-quadruplex recognition specificity shown in RLS mode was even better than in colorimetric and fluorescent modes. To our best acknowledge, this is the first example of G-quadruplex RLS probe. To achieve the specific G-quadruplex-probing in a wide physiological pH range, four cationic porphyrin derivatives (Scheme 1), including three each with four positive charges and an unreported one with eight positive charges, were prepared and selected as potential G-quadruplex RLS probes. The four probes showed their best performance at different pH values, highlighting the important roles of side arm substituents.

## MATERIALS AND METHODS

### Material and reagents

The DNA oligonucleotides, microRNAs (Table 1 and Supplementary Table S1) and calf thymus DNA (CtDNA) were purchased from Sangon Biotech. Co. Ltd (Shanghai, China). The concentrations of the oligonucleotides were represented as single-stranded concentrations. Single-stranded concentration was determined by measuring the absorbance at 260 nm. Molar extinction coefficient was determined using a nearest neighbour approximation (http://www.idtdna.com/analyzer/Applications/OligoAnalyzer), and the calculated molar extinction coefficients were listed in Table 1. The base concentration of CtDNA was determined by the absorbance at 260 nm using the molar absorption coefficient of 6600 M^−1^·cm^−1^. In order to facilitate the comparison with short-stranded oligonucleotides, the CtDNA concentration was also represented as short-stranded concentration (equivalent to a DNA strand with 24 bases). 5,10,15,20-tetrakis(4-hydroxyphenyl)porphyrin (THPP), 1,2-dibromoethane and *N*-(2-hydroxyethyl) piperazine were obtained from TCI Development Co. Ltd (Shanghai, China). TMPipEOPP, m-TPyEOPP and FA-TMPipEOPP were synthesized according to the methods reported by us (41–43). Hydrochloric acid, potassium chloride, disodium ethylenediaminetetraacetic acid (EDTA) and tris(hydroxymethyl)aminomethane (Tris) were obtained from Sigma. *N*,*N*-Dimethylformamide, potassium carbonate, methanol, ethanol, ethyl acetate, dichloromethane, chloroform, and triethylamine were obtained from Jiangtian Co. Ltd (Jiangsu, China). Deionized and sterilized water (resistance > 18 MΩ/cm) was used throughout the experiments. All chemical reagents were of analytical grade and used without further purification.

**Table 1.** The oligonucleotides used in this work

No.	DNA	Sequence (from 5′ to 3′)	Extinction coefficient [L·mol^−1^·cm^−1^]	Structure
**1**	ssDNA1	GAGCTCTCGAAAGAGCTCCGATTA	235800	ssDNA
**2**	ssDNA2	TAGAGCACACCTGTCCGTG	179500	ssDNA
**3**	dsDNA	TAGAGCACACCTGTCCGTGCACGGACAGGTGTGCTCTA	360400	Short dsDNA
**4**	CtDNA			Long dsDNA
**5**	KRAS	AGGGCGGTGTGGGAAGAGGGAAGAGGGGGAGG	341000	Parallel G-quadruplex
**6**	Oxy28	GGGGTTTTGGGGTTTTGGGGTTTTGGGG	101100	Antiparallel G-quadruplex
**7**	Hum24	TTAGGGTTAGGGTTAGGGTTAGGG	244600	Mixed-type G-quadruplex
**8**	C-MYC	TGAGGGTGGGGAGGGTGGGGAA	229900	Parallel G-quadruplex
**9**	CatG4	TGGGTAGGGCGGGTTGGG	176200	Parallel G-quadruplex
**10**	AS1411	GGTGGTGGTGGTTGTGGTGGTGGTGG	250800	Parallel G-quadruplex

### Structural characterization instruments

Nuclear magnetic resonance (NMR) spectra were recorded on Mercury Vx-500 spectrometer operating for ^1^H-NMR and ^13^C-NMR. Chemical shifts in the ^1^H-NMR spectra are reported in ppm relative to the residual hydrogen atoms in the deuterated solvents: *d* = 2.50, 3.30 and 7.25 ppm for _[D6]_DMSO and CDCl_3_, respectively. Fourier transform mass spectrometry (FT-MS) was conducted on a Varian 7.0T FT-MS mass spectrometer. Matrix-assisted laser desorption/ionization time-of-flight mass spectrometry (MALDI-TOF-MS) was conducted on a Bruker Autoflex III TOF/TOF 200 instrument. Fluorescence and RLS spectra were recorded on a SHIMADZU RF-5301PC spectrofluorimeter. UV–vis absorption spectra were measured on a Cary 60 UV–vis spectrophotometer (Agilent Technologies).

### RLS spectroscopy

Solutions containing 10 μM individual oligonucleotides, 20 mM Tris–HCl buffer (at pH of 7.4, 7.0, 6.5, 6.0), 50 mM KCl and 1 mM Na_2_EDTA were prepared. Each solution was heated at 95°C for 5 min, then cooled rapidly to 25°C and was allowed to incubate at this temperature for 30 min. After overnight incubation at 4°C, 5 μM of porphyrin was added. RLS spectra were recorded on a SHIMADZU RF-5301PC spectrofluorimeter with 1 cm-path-length micro quartz cell (40 μl, Starna Brand, England) by synchronously scanning the excitation and emission monochromators at Δλ = 0 nm. When the RLS spectrum in the range of 600–800 nm was investigated, excitation and emission slits were both set at 3 nm. When the RLS spectrum in the range of 250–900 nm was investigated, excitation and emission slits were set at 3 and 1.5 nm, respectively.

RLS titration experiments were carried out by varying the DNA concentration but maintaining the porphyrin concentration at 5 μM. The sample solutions were prepared and detected as aforementioned.

Job plot analysis was performed by systematic variation of the molar fraction of porphyrin and G-quadruplex while keeping a constant total concentration of 10 μM. The mixtures were prepared as above, and the RLS spectra in the range of 600–800 nm were recorded.

### UV–vis absorption spectroscopy

UV–vis absorption spectra were measured on a Cary 60 UV–vis spectrophotometer (Agilent Technologies) with 1cm-path-length microquartz cell (40 μl, Starna Brand, England). The sample solutions were prepared as aforementioned and the absorption spectra in the range of 350–800 nm were recorded. Absorption spectrum titration experiments were carried out by varying the DNA concentration but maintaining the porphyrin concentration at 5 μM.

### Fluorescent spectroscopy

Fluorescence spectra were also measured on a SHIMADZU RF-5301PC spectrofluorimeter with 1 cm-path-length micro quartz cell (40 μl, Starna Brand, England). The sample solutions were prepared as aforementioned. Fixing the excitation wavelength at 454 nm, the fluorescence spectra in the range of 600–850 nm were recorded (excitation slit = emission slit = 3 nm).

### Circular dichroism (CD) spectroscopy

The CD spectra were recorded in the range of 220–350 nm in 1 cm path length cuvette on a BioLogic MOS-45 spectropolarimeter. In one experiment, solutions were prepared in 16 mM Na_2_HPO_4_/NaH_2_PO_4_ buffer (pH 7.0) containing 50 mM KCl, 1 mM Na_2_EDTA, and 0–10 μM of KRAS. Each mixture was heated at 95°C for 5 min and then cooled rapidly to 25°C and incubated at 25°C for 30 min. After overnight incubation at 4°C, 5 μM TMPipEOPP was added. In one other experiment, solutions were prepared in 16 mM Na_2_HPO_4_/NaH_2_PO_4_ buffer (pH 7.0) containing 50 mM KCl, 1 mM Na_2_EDTA, and 5 μM of KRAS. Each mixture was heated at 95°C for 5 min and then cooled rapidly to 25°C and incubated at 25°C for 30 min. After overnight incubation at 4°C, 0–10 μM TMPipEOPP was added. CD spectra of these solutions were averaged from three scans, which were recorded at 100 nm/min with a response time of 1 s and a bandwidth of 0.5 nm.

### Linear discriminate assay

To apply linear discriminate assay (LDA) for identification of the G-quadruplex structure, we combined all of the data obtained from UV–vis spectroscopy, fluorescent spectroscopy and RLS spectroscopy. With those combined data as the training groups, a Matlab program was employed for the LDA analysis, and the matrix which may project the training groups into clusters with maximal separated distance was generated. Based on the calculated score, the tested DNA could be assigned into G-quadruplex or non-G-quadruplex structure.

### MicroRNA Let-7a-sensing assay

3 μM ON1 and 3 μM ON2 were mixed in 20 mM Tris-HCl buffer (pH 7.0) containing 1mM MgCl_2_. The mixture was heated at 95°C for 5 min and then cooled rapidly to 25°C. After incubation at this temperature for 15 min, 30 mM KCl and different concentrations of target microRNA were added. The mixture was incubated at 25°C for another 1 h. Then, 5 μM of porphyrin was added and the RLS spectrum in the range of 600–800 nm was recorded. The RLS intensity ratio at 702 and 652 nm (*I*_702_/*I*_652_) was used as the output signal for target microRNA detection.

## RESULTS AND DISCUSSION

### Synthesis and characterization of octa-cationic porphyrin

**Scheme 2.**
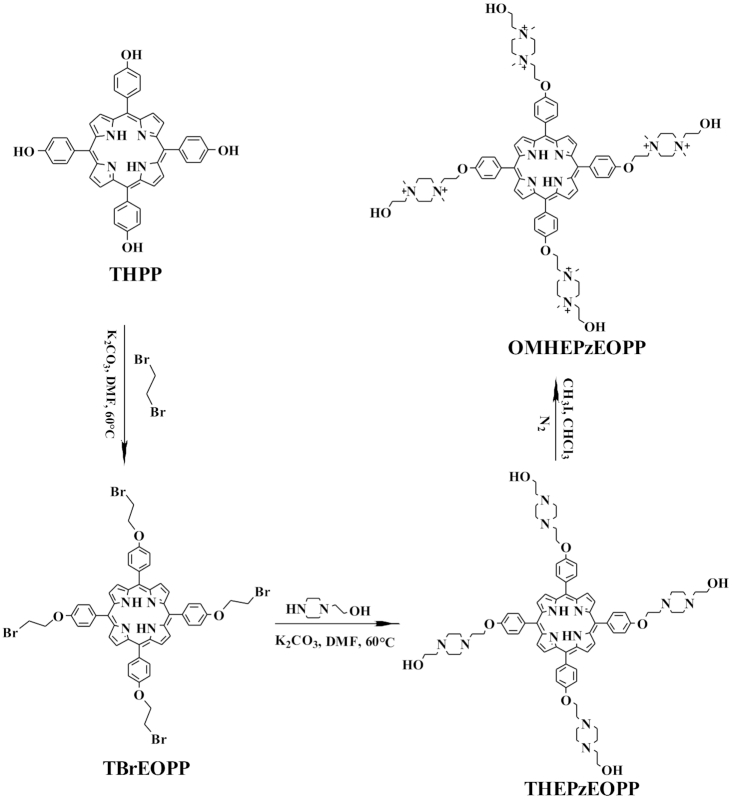
Synthetic route of OMHEPzEOPP.

Four water-soluble cationic porphyrin derivatives, three have four positive charges and one has eight positive charges, were used for the studies on G-quadruplex RLS probes. The three ones with four positive charges have been reported in our previous works (40–44). The one with eight positive charges has never been reported. This octa-cationic porphyrin, named as 5,10,15,20-tetrakis{4-2-[1,4-dimethyl-4-(2-hydroxyethyl)piperazin-1-yl]ethoxy]pheyl}porphyrin (OMHEPzEOPP), containing four piperazine-derivatived side arm substituents each has two positive charges, was synthesized following the synthetic route shown in Scheme 2. TBrEOPP was prepared from THPP according to the method previously reported by our group (42). By elongating the reaction time to 8 h and by optimizing the ratio of column chromatography eluting agent (see supporting information), the TBrEOPP yield was increased to 60%. The obtained TBrEOPP was reacted with N-(2-hydroxyethyl) piperazine to provide the intermediate THEPzEOPP, which was then treated with excess iodomethane to obtain the final product of OMHEPzEOPP. OMHEPzEOPP was well-characterized with ^1^H-nuclear magnetic resonance (^1^H-NMR) spectroscopy and ^13^C-NMR spectroscopy, and the two intermediates of TBrEOPP, THEPzEOPP were all well-characterized with ^1^H-NMR spectroscopy and mass spectroscopy (MS) to confirm the product structure and assure the product purity. The details of porphyrin synthesis and characterization are available in the Supporting Information (Supplementary Figures S1–S7). By comparing the ^1^H-NMR spectra of THEPzEOPP and OMHEPzEOPP, it was found that OMHEPzEOPP showed a new single peak at 3.59 ppm which contained 24 hydrogens, indicating that the nitrogen atoms on the four piperazine rings were successfully linked by methyl groups. The peak at 46.55 ppm in ^13^C-NMR spectrum of OMHEPzEOPP further demonstrated the presence of methyl carbon. These characterizations confirmed the successful synthesis of highly purified octa-cationic porphyrin derivative. Previous studies from our and other groups demonstrated that the presence of positive charges in porphyrin derivatives could promote their binding with DNA through electrostatic interaction (40–43,45,46). Although more and more porphyrin derivatives with positive charges have been reported, the octa-cationic porphyrins were rarely reported. As far as we known, only one octa-cationic porphyrin has been reported before (47), and we give the second example. The synthetic route given in this work could give the octa-cationic porphyrin product with high yield, thus might provide a simple and useful way for the preparation of porphyrins with high charges.

### Colorimetric, fluorescent and RLS recognition of G-quadruplexes

**Figure 1.**
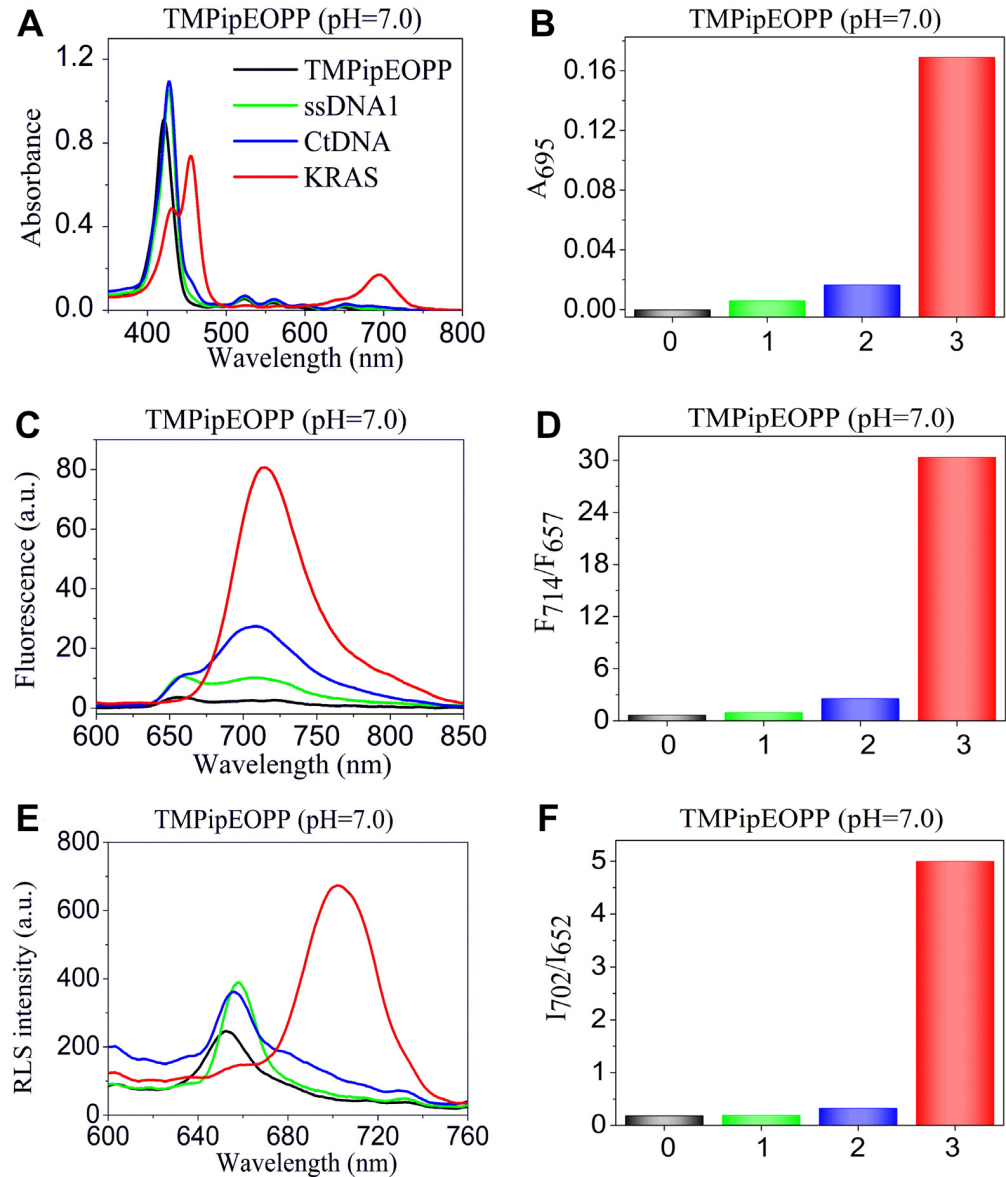
(**A**, **B**) UV-vis absorption, (**C**, **D**) fluorescent, (**E**, **F**) RLS spectra and corresponding signal outputs of TMPipEOPP in the absence or presence of different DNAs at pH of 7.0. Free porphyrin (black line); ssDNA (green line); dsDNA (blue line); G-quadruplex (red line). The effects of other DNAs were shown in Supplementary Figures S8–S11.

In our previous works (40,41), we have demonstrated that increasing the sizes of side arm substituents is a good way to improve the recognition specificity of porphyrins towards G-quadruplexes since the increased steric hindrance will prevent their intercalation into the base pairs of double-stranded DNAs (dsDNAs), and a series of water-soluble cationic porphyrin derivatives were synthesized and demonstrated to work well for colorimetric and fluorescent G-quadruplex-probing under different pH conditions (40–44). Herein, we find that they can also be developed as excellent RLS probes for ratiometric recognition of G-quadruplexes. Figure 1 shows the representative results of the cationic porphyrin of TMPipEOPP. This porphyrin could give distinctly different colorimetric, fluorescent and RLS responses to G-quadruplexes compared to dsDNAs and single-stranded DNAs (ssDNAs). G-quadruplexes could cause obvious hypochromicity of the porphyrin Soret band at around 420 nm (Figure 1A), accompanied by the emergence of two new absorption peaks centered at around 454 and 695 nm respectively. On the contrary, neither dsDNAs nor ssDNAs could cause the appearance of the two new bands. When excited at 454 nm, TMPipEOPP gave two weak fluorescence peaks centered at around 657 and 714 nm, respectively (Figure 1C). With the addition of G-quadruplexes (e.g. KRAS, Table 1), only one strong fluorescence band centered at around 714 nm remained, thus giving a greatly increased F_714_/F_657_ ratio. In the presence of ssDNAs (e.g. ssDNA1) or dsDNA (e.g. calf thymus DNA, CtDNA), however, two fluorescence peaks still could be observed, and corresponding F_714_/F_657_ ratios were much lower than those given by G-quadruplexes. By utilizing the absorbance at 695 nm (Figure 1B) and the fluorescence ratio of F_714_/F_657_ (Figure 1D), specific colorimetric and fluorescent recognition of G-quadruplexes could be achieved by the porphyrin probe, respectively.

By synchronously scanning the excitation and emission monochromators at Δλ = 0 nm, the RLS spectra of porphyrins in the absence or presence of different structural DNAs could be obtained. Figure 1e showed the representative RLS spectra of TMPipEOPP in the range of 600–760 nm. In this wavelength range, free TMPipEOPP showed a RLS peak at around 652 nm, which corresponds to the Q-band absorption of the porphyrin. With the addition of G-quadruplex, the RLS peak at around 652 nm nearly disappeared, accompanied by the emergence of a much stronger RLS peak at around 702 nm, which corresponds to the new peak caused by G-quadruplex in UV–vis absorption spectrum (Figure 1A). By utilizing the changes in RLS signals at these two wavelengths, G-quadruplexes could be easily probed. Different from G-quadruplexes, none of ssDNAs and dsDNAs (including long dsDNA CtDNA) showed obvious effects on the RLS signal of TMPipEOPP. That is, the new RLS peak at 702 nm did not emerge, and the RLS intensity at 702 nm remained much smaller than that at 652 nm. By comparing the RLS intensity ratio of *I*_702_/*I*_652_, G-quadruplexes could be easily discriminated from ssDNAs and dsDNAs (Figure 1F). Collectively, by recording the changes in absorption signal at 695 nm (*A*_695_), fluorescence intensity of F_714_/F_657_ and RLS intensity ratio of *I*_702_/*I*_652_, TMPipEOPP can be used as specific optical probes for colorimetric, fluorescent or RLS recognition of G-quadruplexes.

**Figure 2.**
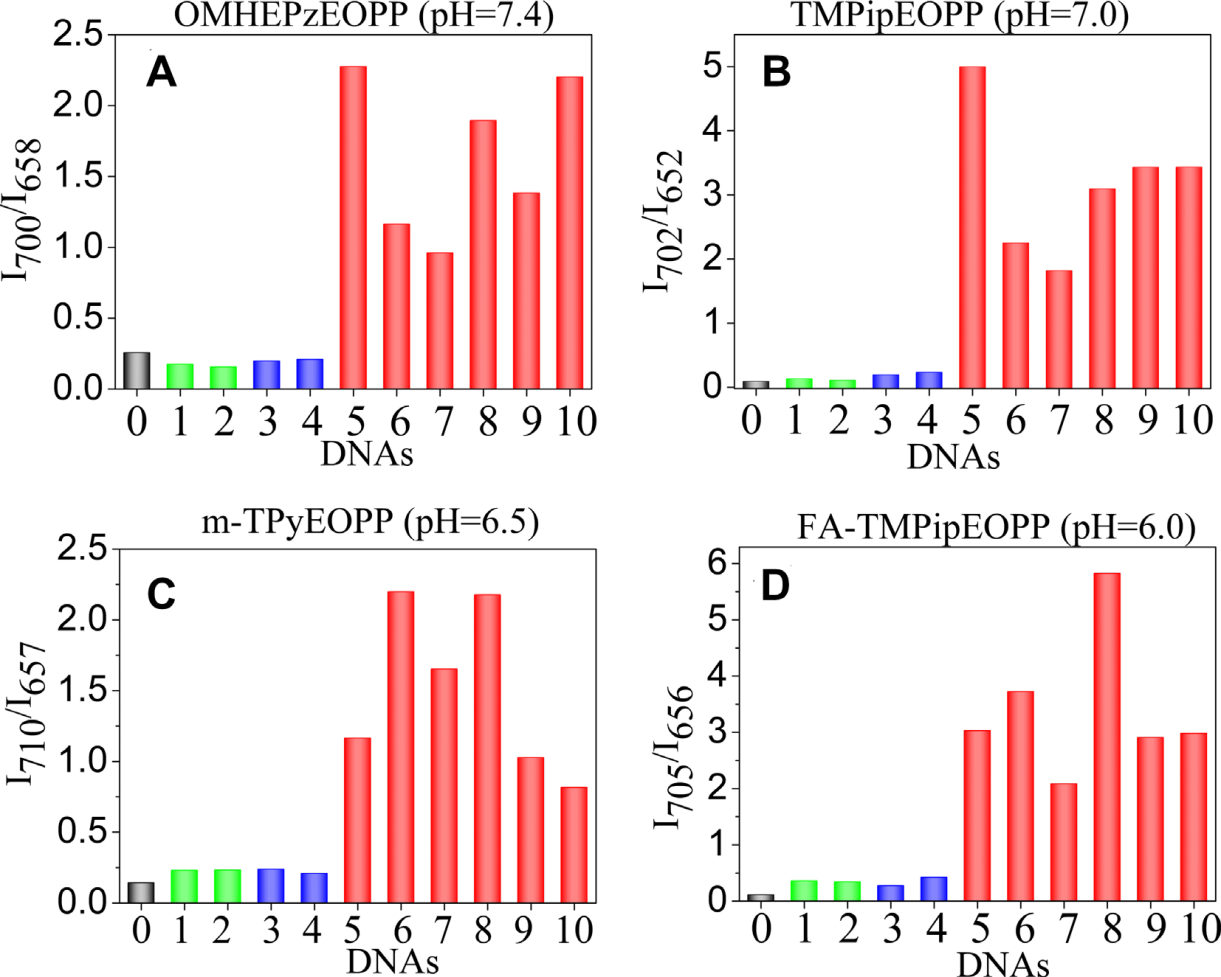
RLS intensity ratio of (**A**) OMHEPzEOPP, (**B**) TMPipEOPP, (**C**) m-TPyEOPP and (**D**) FA-TMPipEOPP in the absence or presence of different structural DNAs at pH 7.4, 7.0, 6.5 and 6.0, respectively. ‘0’ represent free porphyrins, and the serial number of DNA is same to that in Table 1. Free porphyrin (black); ssDNAs (green); dsDNAs (blue); G-quadruplexes (red). [porphyrin] = 5 μM; [DNA] = 10 μM.

To demonstrate the universality of TMPipEOPP as RLS probe for G-quadruplex-specific recognition, six commonly studied G-quadruplexes (Table 1), including parallel (KRAS, C-MYC, CatG4 and AS1411), antiparallel (Oxy28) and parallel/antiparallel-mixed (Hum24) ones were selected and tested. The results (Figure 2b) showed that all of these G-quadruplexes could give obviously higher RLS intensity ratios than ssDNAs and dsDNAs, thus indicating that TMPipEOPP might be used as a universal RLS probe working well for all G-quadruplexes. Herein, the ratio of the RLS intensities at two wavelengths was used as signal output. Due to the self-referencing capability, such a ratiometric measurement modality can give high detection accuracy by reducing the effects of the fluctuation of some factors, including light source intensity, instrument sensitivity and probe concentration (22,48,49).

We have previously found that changing the side arm substituents could endow the cationic porphyrin derivatives with pH-dependent colorimetric and fluorescent recognition behaviors towards G-quadruplexes (42,43). Similarly, as RLS probes, the tested cationic porphyrins also showed pH-dependent G-quadruplex recognition specificity. By carefully comparing the RLS responses of a series of cationic porphyrins towards G-quadruplexes, ssDNAs and dsDNAs, we selected a suitable cationic porphyrin to specifically probe G-quadruplexes for each of four representative pH conditions. That is, OMHEPzEOPP for pH 7.4, TMPipEOPP for pH 7.0, m-TPyEOPP for pH 6.5 and FA-TMPipEOPP for pH 6.0. Under their individual appropriate pH conditions, all of these porphyrins might be developed as universal G-quadrplex-specific RLS probes (Figure 2).

### RLS titration spectra of the four porphyrins

**Figure 3.**
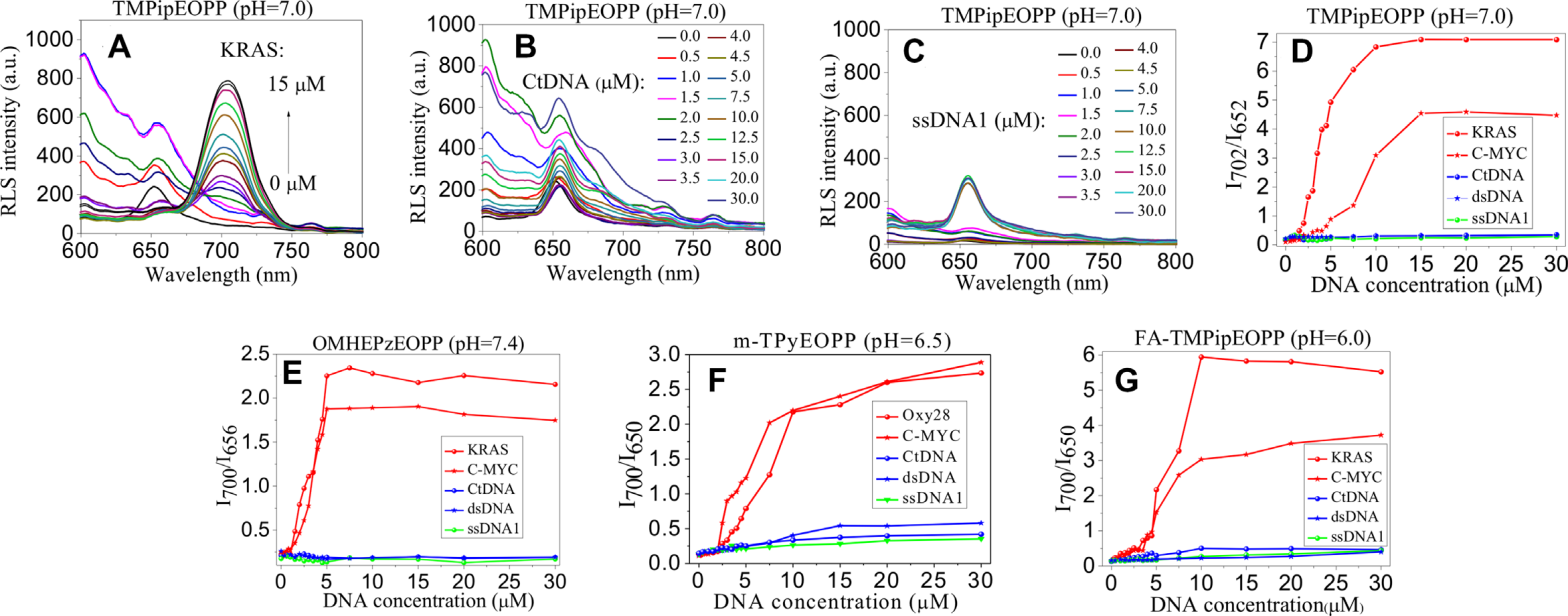
(A–C) DNA concentration-dependent RLS spectral changes of TMPipEOPP. (**A**) KRAS (G-quadruplex); (**B**) CtDNA (dsDNA); (**C**) ssDNA1 (ssDNA). (**D–G**) DNA concentration-dependent RLS intensity ratio changes of the four porphyrin probes under their individual appropriate pH conditions. [porphyrin] = 5 μM. Excitation and emission slits were both set at 3 nm.

To further demonstrate the feasibility of these RLS probes for specific recognition of G-quadruplexes, G-quadruplex concentration-dependent RLS signal changes (Figure 3 and Supplementary Figure S17) were recorded and compared with those induced by ssDNAs and dsDNAs. Figure 3A–C showed the RLS responses of TMPipEOPP to representative G-quadruplex, ssDNA and dsDNA. Addition of G-quadruplex KRAS could result in the emergence of RLS peak at around 702 nm, and its peak intensity continuously increased with G-quadruplex concentration. As a result, a significant DNA concentration-dependent *I*_702_/*I*_652_ ratio change was observed for G-quadruplexes (Figure 3D). On the contrary, addition of ssDNA or dsDNA led to more complex changes in the RLS spectrum of TMPipEOPP, but the RLS intensity at 652 nm was consistently higher than that at 702 nm. Correspondingly, the *I*_702_/*I*_652_ ratio kept at a low level and almost unchanged with ssDNA or dsDNA concentration. These results further confirmed that TMPipEOPP could be used as a specific RLS probe for ratiometric G-quadruplex recognition at pH 7.0. Similar DNA concentration-dependent RLS signal changes were given by OMHEPzEOPP at pH of 7.4 (Supplementary Figure S14), m-TPyEOPP at pH of 6.5 (Supplementary Figure S20) and FA-TMPipEOPP (Supplementary Figure S23) at pH of 6.0, respectively. Collectively, using these four cationic porphyrins as RLS probes, specific G-quadruplex-probing could be achieved in the important physiological pH range of 7.4–6.0.

In the studies on G-quadruplex-probing, highly specific discrimination of G-quadruplexes from long dsDNA is usually a difficult problem. For comparison purposes, DNA concentration-dependent UV-vis (Supplementary Figures S12, S15, S18 and S21) and fluorescence titration spectra (Supplementary Figures S13, S16, S19 and S22) of the four porphyrin derivatives were also given in the Supporting Information. When these porphyrins were used for colorimetric or fluorescent probing G-quadruplexes, weak nonspecific signal responses could also be given by high concentration of long dsDNA (for example CtDNA) though the signals were still distinguishable from those given by G-quadruplexes. Encouragingly, in RLS probing mode, almost no any signal response was given by CtDNA even at high concentration range, these results imply that RLS might have better G-quadruplex-probing specificity against long dsDNAs than colorimetric and fluorescent modes.

### RLS response mechanism of the cationic porphyrins to G-quadruplexes

**Figure 4.**
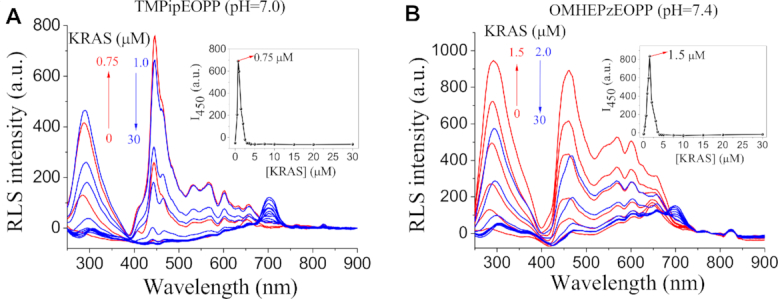
RLS spectra of (**A**) TMPipEOPP and (**B**) OMHEPzEOPP in the presence of different concentrations of KRAS. The RLS intensity increased first (red lines) and then declined (blue lines) with KRAS concentration. The inserts show the KRAS concentration-dependent changes in the RLS signal intensity at 450 nm. [TMPipEOPP] = [OMHEPzEOPP] = 5 μM. Excitation and emission slits were set at 3 and 1.5 nm, respectively.

To elucidate the RLS signal generation mechanism of the four porphyrins, DNA concentration-dependent porphyrin RLS spectral changes were recorded in a wide wavelength range of 250–900 nm. Similar spectral change tendencies were given by the four cationic porphyrins. Figure 4 showed the representative spectral changes of TMPipEOPP and OMHEPzEOPP caused by KRAS. With the increase of KRAS concentration, the biggest RLS spectral changes were observed at around 450 nm. The RLS intensity at this wavelength sharply increased with KRAS concentration from 0 to 0.75 μM and 0 to 1.5 μM for TMPipEOPP and OMHEPzEOPP, respectively, and then significantly decreased with further addition of KRAS. Addition of dsDNA or ssDNA could also lead to similar signal changes in this spectral range (Supplementary Figures S24–S27). Circular dichroism (CD) analysis suggested that the addition of TMPipEOPP had no effects on the conformation of G-quadruplex, and G-quadruplex conformation did not change with DNA concentration (Supplementary Figure S28). Thus, the RLS signal changes in this spectral range might be attributed to the aggregation of positively-charged porphyrin along negatively-charged DNA strand. When DNA/porphyrin concentration ratio is low, J-type aggregates might be formed by porphyrin with the assistance of DNAs. With the increase of DNA/porphyrin concentration ratio, the aggregates were dissolved due to the dispersion of porphyrin by more DNA strands. Different from the trend of descending after ascending showed at around 450 nm, the RLS signal at around 702 nm continuously increased with G-quadruplex concentration. In addition, only G-quadruplex but not ssDNA and dsDNA could induce the emergence of the RLS peak at this wavelength. These results suggested that the RLS spectral changes in these two wavelength ranges might be caused by different mechanisms. That is, the signal change at around 702 nm is irrelevant with porphyrin aggregation, but might be attributed to the specific interaction between porphyrin and G-quadruplexes. Taking TMPipEOPP and OMHEPzEOPP as examples, Job plot analysis based on the RLS signal intensity ratio gave a 1:1 binding stoichiometry for both of them to KRAS (Supplementary Figure S29), thus suggesting they might interact with KRAS by an end-stacking mode. Since high density of negative charges of G-quadruplex can promote the gathering of H^+^ ions around DNA surface, such a binding mode can then lead to the N-protonation of TMPipEOPP (43,50), thus giving the emergence of absorption peak at 695 nm in UV-vis spectrum and RLS peak at 702 nm in RLS spectrum. Collectively, the RLS signal changes in the low wavelength range are caused by DNA-assisted porphyrin aggregation, but the signal changes in the high wavelength range that we used for specific G-quadruplex-probing are related to the porphyrin protonation promoted by end-stacking of porphyrin on G-quadruplexes. OMHEPzEOPP has eight positive charges, which might promote the end-stacking of OMHEPzEOPP on G-quadruplexes due to the increased contribution of electrostatic interaction, thus could overcome the adverse effects of decreased H^+^ concentration on porphyrin protonation at high pH range. Therefore, OMHEPzEOPP can work for RLS probing of G-quadruplexes at a relatively high pH condition (e.g. pH 7.4). Compared with TMPipEOPP, m-TPyEOPP has four pyridinium moieties in its side arms, the positive charge delocalization of peripheral pyridinium moieties on the porphyrin macrocycle would increase the difficulty of N-protonation of porphyrin core to some extent (51), which makes m-TPyEOPP show a better G-quadruplex recognition specificity than TMPipEOPP at a relatively acidic condition (e.g. pH 6.5) (42). Different from other three porphyrin derivatives, FA-TMPipEOPP has an asymmetrical molecular structure, which is benefit for neither DNA-assisted aggregation nor end-stacking on G-quadruplex. However, such a decreased DNA-binding stability makes it suitable for specific G-quadruplex-probing at low pH range (e.g. pH 6.0) in which H^+^ is abundant. No similar ratiometric RLS signal changes were observed for some reported G-quadruplex probes (e.g. thiofavin T, crystal violet and malachite green, Supplementary Figure S30) (52–54), confirming the unique N-protonation of cationic porphyrins.

### Distinguishing G-quadruplex by linear discriminant analysis

**Figure 5.**
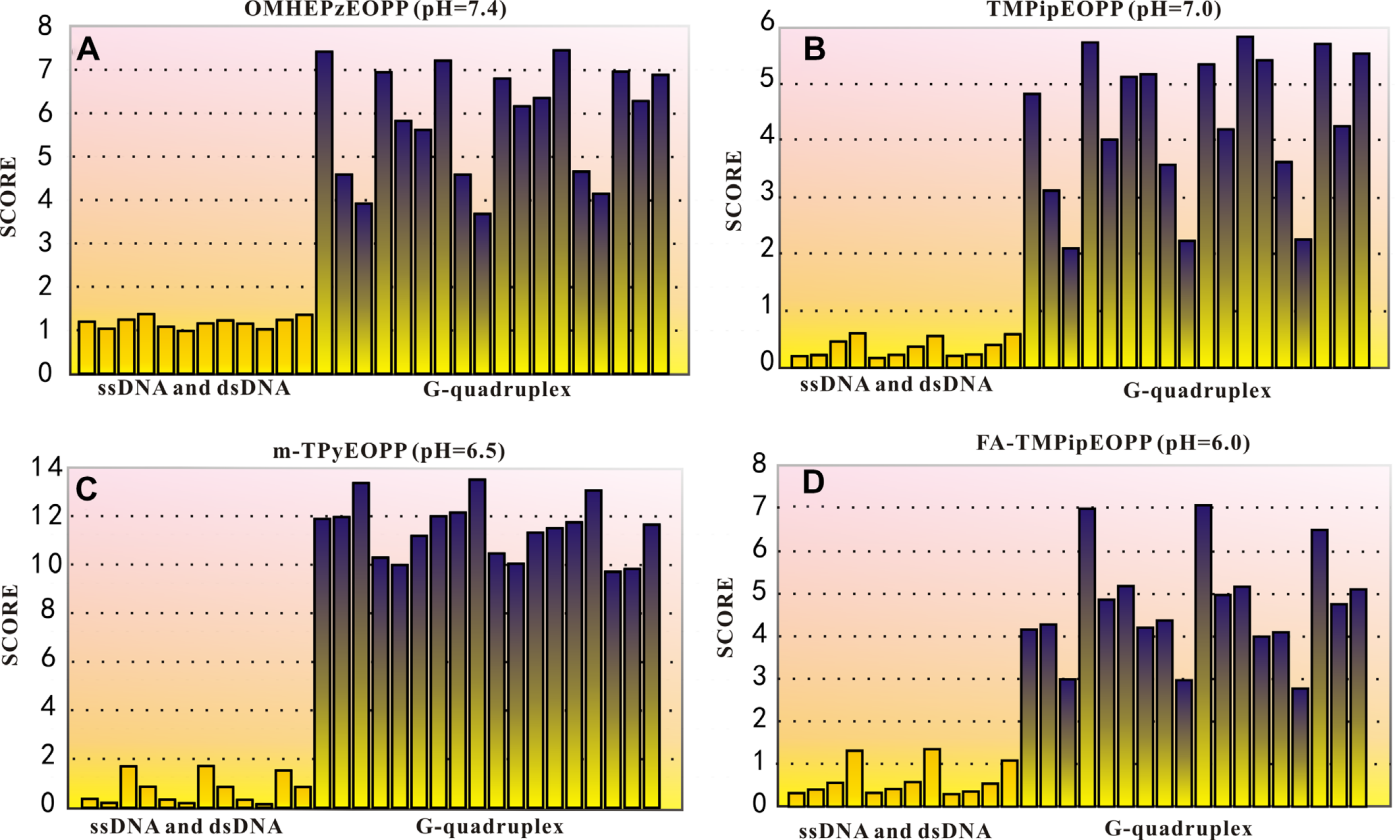
The result of LDA for (**A**) OMHEPzEOPP, (**B**) TMPipEOPP, (**C**) m-TPyEOPP and (**D**) FA-TMPipEOPP in the absence or presence of different structural DNAs at pH 7.4, 7.0, 6.5 and 6.0, respectively. ssDNA and dsDNA (yellow); G-quadruplex (purple).

Since the prepared cationic porphyrins with large side substituents could simultaneously provide three ways (UV-vis absorption, fluorescent and RLS) to specifically recognize G-quadruplexes, we could then apply a statistical method—linear discriminant analysis (LDA) to achieve a quantifiable discrimination of G-quadruplexes from ssDNAs and dsDNAs. LDA is a statistical method which is widely applied to assign new objects to known classes or separate classes of objects (55,56). Briefly, the measured variables, such as the different spectra obtained by UV-vis, fluorescence or RLS, were first combined and set as original matrix. The linear combinations of the original matrix were obtained as discriminants. Discriminant functions are calculated with the objective of maximizing the distance between different classes. Finally, treating with the obtained discriminant function, the original matrix was converted into a series of scores accorded with the appropriate objects. The scores were applied to value the difference between different classes of objects. As the result shown in Figure 5, the scores between ssDNA or dsDNA and G-quadruplex showed great differences. We set a threshold at 2.0, the DNA with a calculated value higher than this threshold could be definitely classified as G-quadruplex, otherwise is assigned to other structural DNA. Because this statistical method takes into account the results of UV–vis absorption, fluorescent and RLS analysis, not only a quantifiable but also a more credible judgment can be given for G-quadruplex-probing.

### Biosensing application of RLS probes

Excellent G-quadruplex recognition specificity shown by the tested cationic porphyrins makes them promising in biosensing applications. More interestingly, we found that the G-quadruplex-probing performance of the proposed RLS probes could be further improved by adding non-G-quadruplex DNAs, especially in the low G-quadruplex concentration range. For example, in the presence of 5 μM ssDNA1, TMPipEOPP could give obvious RLS response to 100 nM KRAS (Supplementary Figure S31). In the absence of ssDNA1, however, RLS signal output distinguishable from the blank control could be observed only when the KRAS concentration was increased to 1 μM. This observation can be reasonably explained by the proposed working mechanism. In the absence of ssDNA1, addition of low concentrations of KRAS leads to the aggregation of TMPipEOPP along DNA strands, resulting in the increase of RLS intensity at both 652 and 702 nm, which will mask the RLS response (*I*_702_/*I*_652_) to KRAS. Addition of ssDNA1 can destroy the DNA-assisted TMPipEOPP aggregation. As a result, interference from aggregation will be efficiently overcome. Such a finding makes the proposed RLS probes more suitable for biosensing applications since many G-quadruplex-based sensing operations are related with G-quadruplex formation or destruction and the presence of irrelevant DNAs is usually inevitable. Under the same conditions, addition of irrelevant DNAs showed no improvement for fluorescent and colorimetric sensing of G-quadruplexes. In the presence of 5 μM ssDNA1, distinguishable single outputs could be given when 200 and 600 nM KRAS was added in fluorescent and colorimetric modes, respectively (Supplementary Figure S32).

**Figure 6.**
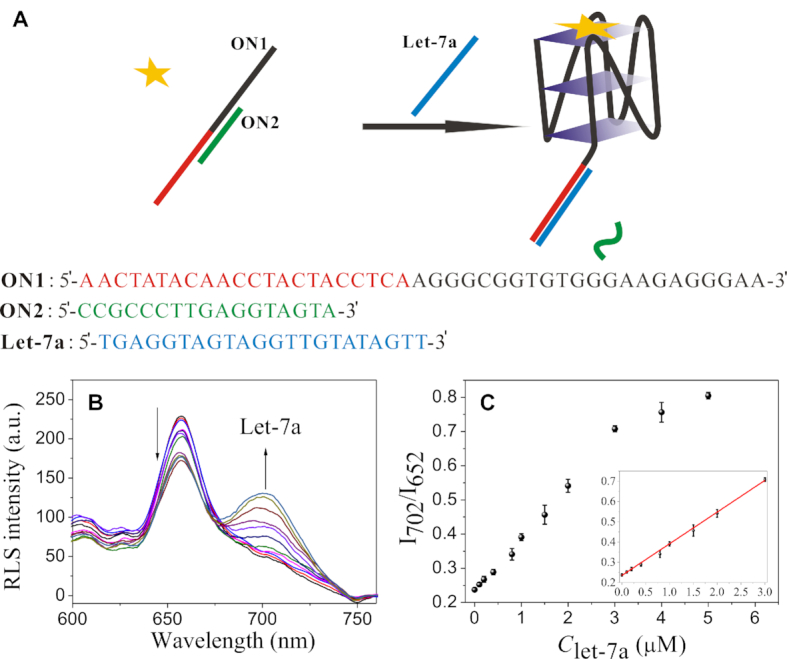
Let-7a detection using TMPipEOPP as RLS probe. (**A**) Working mechanism of the sensing platform. (**B**) RLS spectra of the sensing solutions in the presence of different concentrations of Let-7a. (**C**) Let-7a concentration-dependent changes in RLS intensity ratio of *I*_702_/*I*_652_. The insert shows the linear relationship between *I*_702_/*I*_652_ value and Let-7a concentration in the range of 0–3.0 μM. [TMPipEOPP] = 5 μM.

We next investigated the potential of this kind of RLS probes for biosensing applications. As a proof-of-concept example, its feasibility for label-free detection of microRNA in aqueous solution was tested. To achieve this, two DNA oligonucleotides were prepared. The longer one (ON1) is consisted of two parts (Figure 6A): G-rich sequence with G-quadruplex-forming potential and probing sequence that is complimentary to target microRNA (Let-7a). The shorter one (ON2) is complimentary to the middle part of ON1, and the formation of ON1/ON2 duplex can hamper the formation of G-quadruplex by ON1, which was reflected by the low *I*_702_/*I*_652_ value given by the RLS probe of TMPipEOPP. With the addition of target Let-7a, ON2 dissociated from ON1 via a toehold-mediated strand displacement reaction. The released G-rich sequence could fold into G-quadruplex structure, which was probed by TMPipEOPP to give increased RLS signal (Figure 6B). As shown in Figure 6C, the *I*_702_/*I*_652_ value continuously increased with Let-7a concentration, and a linear relationship was obtained in the concentration range of 100 nM to 3 μM. Based on the rule of 3σ/S, the detection limit was calculated to be 22 nM. Considering that no any signal amplification step is used in this sensing system, such a detection limit is acceptable (57–59). Using OMHEPzEOPP as RLS probe, similar results could be given at pH of 7.4 (Supplementary Figure S33). The sensing system also gave good specificity, and obviously different output signals were given by perfectly matched target Let-7a and mismatched targets, including single-base mismatched one Let-7g (Supplementary Figure S34).

## CONCLUSIONS

In summary, four water-soluble cationic porphyrins with large side arm substituents, including one with eight positive charges that has never been reported, were prepared and demonstrated to work well for specific G-quadruplex-probing under their individual suitable pH conditions via either individual UV–vis absorption, fluorescent, RLS modes or the three modes-combined statistical method LDA. As a new G-quadruplex-probing mode, the proposed RLS-based one showed excellent specificity. Its G-quadruplex recognition specificity against long dsDNA was even better than corresponding colorimetric and fluorescent modes. As a proof-of-concept, such a RLS probe was demonstrated to work well for label-free and sequence-specific sensing of microRNA. To our best knowledge, this is the first example of RLS G-quadruplex probe. It will greatly expand the family of G-quadruplex probes. As a new member of this family, RLS probe might provide some different information from that given by other probes, for example, the information related with changes in target size, shape and aggregation (60). This work also provides a simple and useful way for the preparation of porphyrins with high charges.

## Supplementary Material

Supplementary Data
