# Supplementary material for: Cationic porphyrins with large side arm substituents as resonance light scattering ratiometric probes for specific recognition of nucleic acid G-quadruplexes
Source: Nucleic Acids Res. 2019 Feb 4;47(6):2727–38. doi: 10.1093/nar/gkz064 (PMC6451126; doi:10.1093/nar/gkz064)
Supplement: Supplementary Data [file gkz064_supplemental_files.docx]

**SUPPLEMENTARY DATA**

**Cationic porphyrins with large side arm substituents as resonance light scattering ratiometric probes for specific recognition of nucleic acid G-quadruplexes**

Li-Ming Zhang^1^, Yun-Xi Cui^2^, Li-Na Zhu^1,^*, Jun-Qing Chu^1^ and De-Ming Kong^2^

^1^ Department of Chemistry, School of Science, Tianjin University, Tianjin, 300072, China

^2^ Tianjin Key Laboratory of Biosensing and Molecular Recognition, College of Chemistry, Nankai University, Tianjin, 300071, China

* Corresponding to: Li-Na Zhu. Tel: +86-13132282917; Fax: +86-22-27403475; E-mail: [linazhu@tju.edu.cn](mailto:linazhu@tju.edu.cn).

Present Address: [Li-Na Zhu], Department of Chemistry, School of Science, Tianjin University, Tianjin, 300072, China

Dedicated to the 80th Birthday of Prof. Dai-Zheng Liao

1. **Experimental details** S-1

**1.1. Synthesis and characterization of OMHEPzEOPP**  S-1

**1.1.1 Synthesis of 5,10,15,20-tetrakis[4-(2-bromoethoxy)phenyl]porphyrin**

**(TBrEOPP)** S-1

**1.1.2 Synthesis of 5,10,15,20-tetrakis{4-2-[4-(2-hydroxyethyl)piperazin-1-yl]**

**ethoxy]phenyl}porphyrin (THEPzEOPP)** S-2

- - 1. **Synthesis of 5,10,15,20-tetrakis{4-2-[1,4-dimethyl-4-(2-hydroxyethyl) piperazin-1-yl]ethoxy]phenyl}porphyrin (OMHEPzEOPP)** S-4

1. **Colorimetric, fluorescent and RLS recognition of G-quadruplex** S-6

**2.1. OMHEPzEOPP at pH 7.4** S-6

**2.2. TMPipEOPP at pH 7.0** S-7

**2.3. m**-**TPyEOPP at pH 6.5** S-8

**2.4. FA**-**TMPipEOPP at pH 6.0**S-9

1. **UV-vis, fluorescent and RLS titration spectra** S-10

**3.1. OMHEPzEOPP at pH 7.4** S-10

**3.1.1 UV**-**vis titration spectra** S-10

**3.1.2 Fluorescent titration spectra** S-10

**3.1.3 RLS titration spectra** S-11

**3.2. TMPipEOPP at pH 7.0** S-11

**3.2.1 UV**-**vis titration spectra** S-11

**3.2.2 Fluorescent titration spectra** S-12

**3.2.3 RLS titration spectra** S-12

**3.3. m-TPyEOPP at pH 6.5** S-13

**3.3.1 UV**-**vis titration spectra** S-13

**3.3.2 Fluorescent titration spectra** S-13

**3.3.3 RLS titration spectra** S-14

**3.4. FA**-**TMPipEOPP at pH 6.0** S-14

- - 1. **UV-vis titration spectra** S-14

**3.4.2 Fluorescent titration spectra** S-15

**3.4.3 RLS titration spectra** S-15

1. **Aggregation behaviors of porphyrins in the presence of DNA** S-16

**4.1. OMHEPzEOPP at pH 7.4** S-16

**4.2. TMPipEOPP at pH 7.0** S-17

**4.3. m**-**TPyEOPP at pH 6.5** S-18

**4.4. FA**-**TMPipEOPP at pH 6.0** S-19

1. **Circular dichroism (CD) analysis of the TMPipEOPP/KRAS mixture**S-20
2. **Job Plot analysis for the binding interaction between OMHEPzEOPP or TMPipEOPP and KRAS** S-21
3. **RLS titration spectra of other G-quadruplex probes**S-22
4. **Improvement of RLS-based G-quadruplex-probing by addition of non-G-quadruplex DNAs**S-23
5. **Fluorescent and colorimetric G-quadruplex-probing in the presence of irrelevant DNAs**S-24
6. **Let-7a-sensing using RLS probes** S-25

**10.1. Oligonucleotides used in microRNA-sensing assay** S-25

**10.2. Let**-**7a**-**sensing using OMHEPzEOPP as RLS probe** S-25

**10.3. Specificity of Let**-**7a assay**S-26

1. **Experimental details**

**1.1. Synthesis and characterization of OMHEPzEOPP**

**1.1.1 Synthesis of 5,10,15,20-tetrakis[4-(2-bromoethoxy)phenyl]porphyrin (TBrEOPP)**

To obtain TBrEOPP, 5,10,15,20-tetrakis(4-hydroxyphenyl)porphyrin (THPP) (0.1573 g, 0.23 mmol) and K_2_CO_3_ (5.00 g, 36.18 mmol) were mixed in 40 mL DMF. Then, 1,2-dibromoethane (2 mL, 23 mmol) in DMF (10 mL) was added dropwise to the mixture under stirring at ambient temperature. The mixture was then stirred at 60 °C for 8 h. After cooling to ambient temperature, the mixture was filtrated under reduced pressure. The crude sample was dissolved in 60 mL CH_2_Cl_2_ and washed with water for five times. The obtained crude product was isolated by silica gel columns (200-300 mesh) using CH_2_Cl_2_/CH_3_CH_2_OH mixture (v:v = 5:1) as the eluant. The red-purple solid of TBrEOPP was obtained in 60% yield (153 mg, 0.138 mmol). ^1^H-NMR (500 MHz, _[D6]_DMSO, 25 °C, TMS, Figure S1) δ (ppm): -2.91 (s, 2H; H-a), 8.87 (s, 8H; H-b), 8.14 (s, 8H; H-c), 7.42 (s, 8H; H-d), 4.63 (s, 8H; H-e), 4.03 (s, 8H; H-f). FT-MS (Figure S2): m/z calcd for [C_52_H_42_Br_4_N_4_O_4_ + H], 1106.55; found 1106.9982 [M + H].

**Figure S1.** ^1^H-NMR of TBrEOPP in (CD_3_)_2_SO.


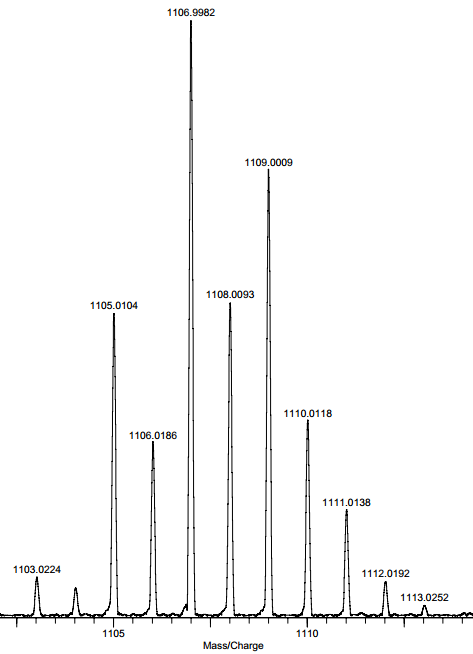

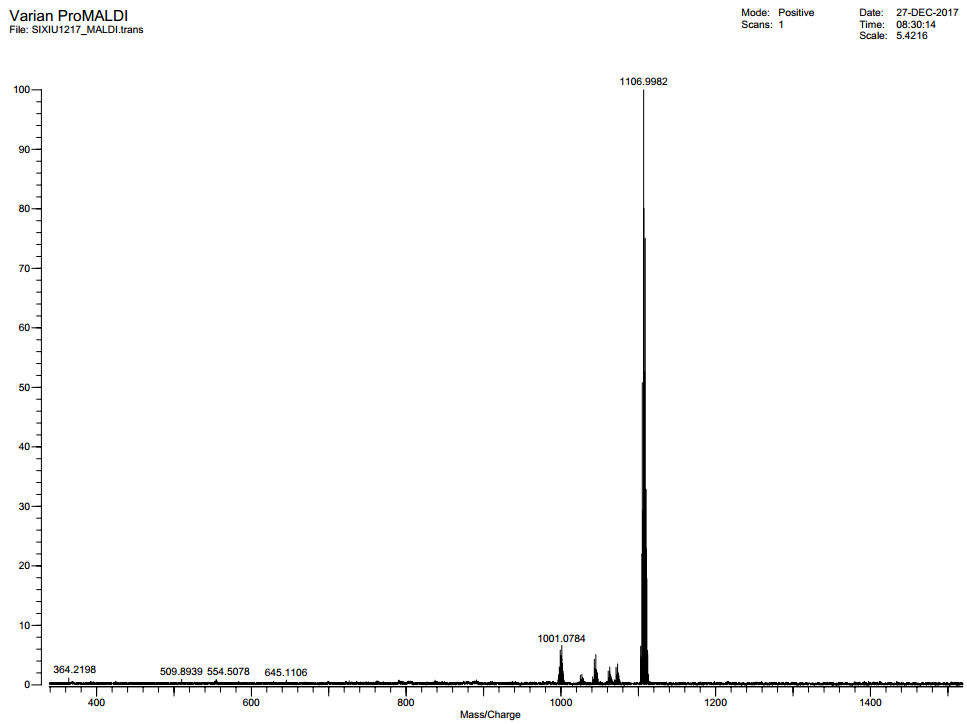


**Figure S2.** FT-MS of TBrEOPP.

**1.1.2 Synthesis of 5,10,15,20-tetrakis{4-2-[4-(2-hydroxyethyl)piperazin- 1-yl]ethoxy]phenyl}porphyrin (****THEPzEOPP)**

N-(2-hydroxyethyl)piperazine (2.5 mL, 20.0 mmol) and K_2_CO_3_ (2.764 g, 20.0 mmol) were mixed in 40 mL DMF. TBrEOPP (0.1120 g, 0.1 mmol) in DMF (10 mL) was added dropwise to this mixture, the mixture was stirred under N_2_ and heated by using an oil bath at 60 °C for 72 h. Then the mixture was filtered under reduced pressure. The filtrate was dissolved in 50 mL CH_2_Cl_2_ and washed with water for five times. After evaporating the organic phase, a red-purple solid was obtained. The crude product was purified by chromatography on silica using CH_2_Cl_2_/CH_3_OH /(CH_3_CH_2_)_3_N mixture (v:v:v=7:1:0.5) as the eluant. The red-purple solid of THEPzEOPP was obtained in 23.0% yield (30 mg, 0.023 mmol). ^1^H-NMR (500 MHz, CDCl_3_, 25 °C, TMS, δ (ppm) (Figure S3): -2.84 (s, 2H; H-a), 8.78 (s, 8H; H-b), 8.04, 8.01 (d, 8H; H-c), 7.21, 7.18 (d, 8H; H-d), 4.33, 4.31, 4.30 (t, 8H; H-e), 2.96, 2.94, 2.93 (t, 8H; H-f), 2.70, 2.61 (d, 32H; H-g), 3.61, 3.59, 3.58 (t, 8H; H-h), 2.56, 2.55, 2.54 (t, 8H; H-i). MALDI-TOF-MS (Figure S4): m/z calcd for [C_76_H_94_N_12_O_8_ + H], 1303.64; found 1303.459 [M + H]; FT-MS (Figure S5): m/z calcd for [C_76_H_94_N_12_O_8_ + H], 1303.64; found 1303.7395 [M + H].

**Figure S3.** ^1^H-NMR of THEPzEOPP in CDCl_3_.

**Figure S4.** MALDI-TOF-MS of THEPzEOPP.


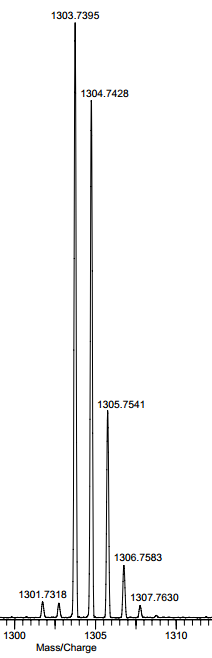

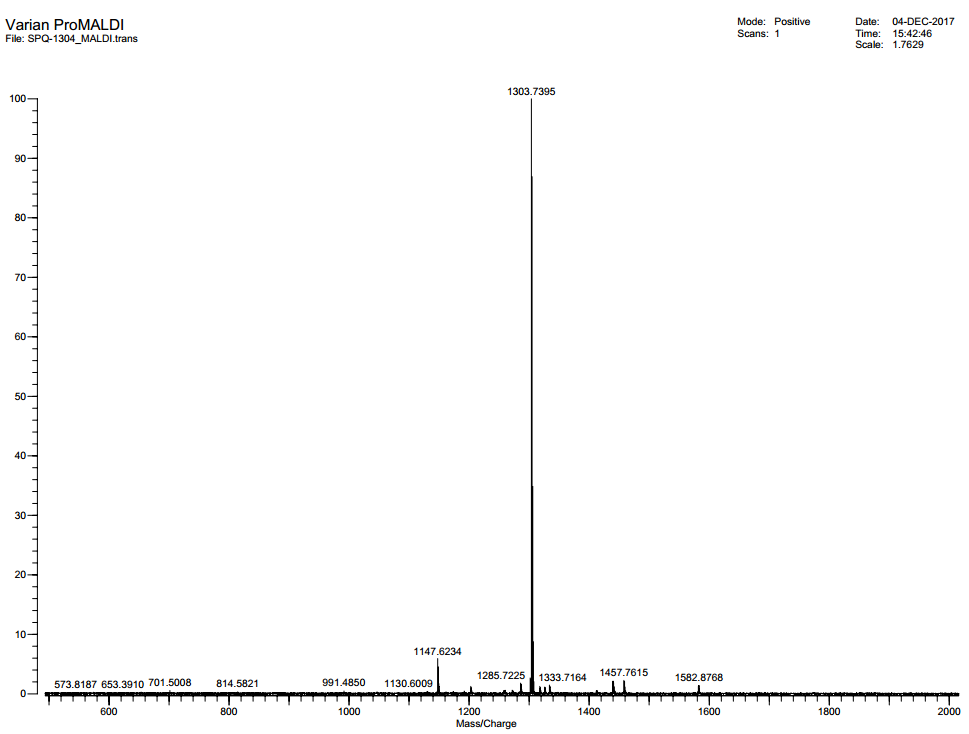


**Figure S5.** FT-MS of THEPzEOPP.

**1.1.3 Synthesis of 5,10,15,20-tetrakis{4-2-[1,4-dimethyl-4-(2-hydroxyethyl) piperazin-1-yl]ethoxy]phenyl}porphyrin (OMHEPzEOPP)**

CH_3_I (10 mL, 0.16 mol) was dissolved in CHCl_3_ (5 mL), and was added dropwise in a solution of THEPzEOPP (0.06 g, 0.046 mmol) in dry CHCl_3_ (35 mL). The mixture was stirred under N_2_ and heated by using an oil bath at 40 °C for 72 h. Then, the reaction mixture was filtered and the resulting solid was washed with CHCl_3_ and diethyl ether in turn. OMHEPzEOPP was obtained as a red purple solid in 60.9% yield (40 mg, 0.028 mmol). ^1^H-NMR (500 MHz, _[D6]_DMSO, 25 °C, TMS) δ (ppm) (Figure S6): -2.89 (s, 2H; H-a), 8.88 (s, 8H; H-b), 8.21 (s, 8H; H-c), 7.50 (s, 8H; H-d), 5.53 (s, 8H; H-e), 4.35 (s, 8H; H-f), 4.15, 4.08, 4.02 (t, 32H; H-g), 4.87 (s, 8H; H-h), 3.88 (s, 8H; H-i ), 3.59 (s, 24H; H-j). ^13^C-NMR (125 MHz, _[D6]_DMSO, 25 °C, TMS, Figure S7) δ (ppm): 120.05 (C-a), 157.54 (C-b), 136.03 (C-c), 135.93 (C-d), 134.78 (C-e), 113.85 (C-f), 157.58 (C-g), 54.87(C-h), 55.12 (C-i), 54.67 (C-j), 60.76 (C-k), 61.82 (C-l), 46.55 (C-m), 53.96 (C-CH_2_Cl_2_).

 **Figure S6.** ^1^H-NMR of OMHEPzEOPP in (CD_3_)_2_SO.


**Figure S7.** ^13^C-NMR of OMHEPzEOPP in (CD_3_)_2_SO.

1. **Colorimetric, fluorescent and RLS recognition of G-quadruplex**

**2.1．OMHEPzEOPP at pH 7.4**

**Colorimetric**

**Fluorescent**

**RLS**

**Figure S8.** (a) UV-vis absorption, (c) fluorescent and (e) RLS spectra of OMHEPzEOPP in the absence or presence of different DNAs. (b) Absorption signal at 700 nm (A_700_), (d) fluorescent intensity ratio (F_713_/F_658_) and (f) RLS intensity ratio (I_700_/I_658_) of OMHEPzEOPP in the absence or presence of different DNAs. The serial number of DNA is same to that in Table 1. Free porphyrin (black); ssDNA (green); dsDNA (blue); G-quadruplex (red). [OMHEPzEOPP] = 5 μM; [DNA] = 10 μM.

- 1. **TMPipEOPP at pH 7.0**

**Colorimetric**

**Fluorescent**

**RLS**

**Figure S9.** (a) UV-vis absorption, (c) fluorescent and (e) RLS spectra of TMPipEOPP in the absence or presence of different DNAs. (b) Absorption signal at 695 nm (A_695_), (d) fluorescent intensity ratio (F_713_/F_658_) and (f) RLS intensity ratio (I_702_/I_652_) of TMPipEOPP in the absence or presence of different DNAs. The serial number of DNA is same to that in Table 1. Free porphyrin (black); ssDNA (green); dsDNA (blue); G-quadruplex (red). [TMPipEOPP] = 5 μM; [DNA] = 10 μM.

- 1. **m-TPyEOPP at pH 6.5**

**Colorimetric**

**Fluorescent**

**RLS**

**Figure S10.** (a) UV-vis absorption, (c) fluorescent and (e) RLS spectra of m-TPyEOPP in the absence or presence of different DNAs. (b) Absorption signal at 700 nm (A_700_), (d) fluorescent intensity ratio (F_725_/F_660_) and (f) RLS intensity ratio (I_710_/I_657_) of m-TPyEOPP in the absence or presence of different DNAs. The serial number of DNA is same to that in Table 1. Free porphyrin (black); ssDNA (green); dsDNA (blue); G-quadruplex (red). [m-TPyEOPP] = 5 μM; [DNA] = 10 μM.

- 1. **FA-TMPipEOPP at pH 6.0**

**Colorimetric**

**Fluorescent**

**RLS**

**Figure S11.** (a) UV-vis absorption, (c) fluorescent and (e) RLS spectra of FA-TMPipEOPP in the absence or presence of different DNAs. (b) Absorption signal at 700 nm (A_700_), (d) fluorescent intensity ratio (F_714_/F_658_) and (f) RLS intensity ratio (I_705_/I_656_) of FA-TMPipEOPP in the absence or presence of different DNAs. The serial number of DNA is same to that in Table 1. Free porphyrin (black); ssDNA (green); dsDNA (blue); G-quadruplex (red). [FA-TMPipEOPP] = 5 μM; [DNA] = 10 μM.

**3. UV-vis, fluorescent and RLS titration spectra**

**3.1. OMHEPzEOPP at pH 7.4**

**3.1.1 UV-vis titration spectra**

**Figure S12.** (a-e) DNA concentration-dependent absorption spectral changes of OMHEPzEOPP under the pH condition of 7.4. (a) KRAS (G-quadruplex); (b) C-MYC (G-quadruplex); (c) CtDNA (dsDNA); (d) dsDNA (dsDNA); (e) ssDNA1 (ssDNA). (f) DNA concentration-dependent A_695_ changes. [OMHEPzEOPP] = 5 μM.

**3.1.2 Fluorescent titration spectra**

**Figure S13.** (a-e) DNA concentration-dependent fluorescence spectral changes of OMHEPzEOPP (λ_ex_ = 454 nm) under the pH condition of 7.4. (a) KRAS (G-quadruplex); (b) C-MYC (G-quadruplex); (c) CtDNA (dsDNA); (d) dsDNA (dsDNA); (e) ssDNA1 (ssDNA). (f) DNA concentration-dependent F_700_/F_650_ changes. [OMHEPzEOPP] = 5 μM.

**3.1.3 RLS titration spectra**

**Figure S14.** (a-e) DNA concentration-dependent RLS spectral changes of OMHEPzEOPP under the pH condition of 7.4. (a) KRAS (G-quadruplex); (b) C-MYC (G-quadruplex); (c) CtDNA (dsDNA); (d) dsDNA (dsDNA); (e) ssDNA1 (ssDNA). (f) DNA concentration-dependent I_700_/I_656_ changes. [OMHEPzEOPP] = 5 μM.

**3.2. TMPipEOPP at pH 7.0**

**3.2.1 UV-vis titration spectra**

**Figure S15.** (a-e) DNA concentration-dependent absorption spectral changes of TMPipEOPP under the pH condition of 7.0. (a) KRAS (G-quadruplex); (b) C-MYC (G-quadruplex); (c) CtDNA (dsDNA); (d) dsDNA (dsDNA); (e) ssDNA1 (ssDNA). (f) DNA concentration-dependent A_695_ changes. [TMPipEOPP] = 5 μM.

**3.2.2 Fluorescent titration spectra**

**Figure S16.** (a-e) DNA concentration-dependent fluorescence spectral changes of TMPipEOPP (λ_ex_ = 454 nm) under the pH condition of 7.0. (a) KRAS (G-quadruplex); (b) C-MYC (G-quadruplex); (c) CtDNA (dsDNA); (d) dsDNA (dsDNA); (e) ssDNA1 (ssDNA). (f) DNA concentration-dependent F_714_/F_657_ changes. [TMPipEOPP] = 5 μM.

**3.2.3 RLS titration spectra**

**Figure S17.** (a-e) DNA concentration-dependent RLS spectral changes of TMPipEOPP under the pH condition of 7.0. (a) KRAS (G-quadruplex); (b) C-MYC (G-quadruplex); (c) CtDNA (dsDNA); (d) dsDNA (dsDNA); (e) ssDNA1 (ssDNA). (f) DNA concentration-dependent I_702_/I_652_ changes. [TMPipEOPP] = 5 μM.

**3.3. m-TPyEOPP at pH 6.5**

**3.3.1 UV-vis titration spectra**

**Figure S18.** (a-e) DNA concentration-dependent absorption spectral changes of m-TPyEOPP under the pH condition of 6.5. (a) Oxy28 (G-quadruplex); (b) C-MYC (G-quadruplex); (c) CtDNA (dsDNA); (d) dsDNA (dsDNA); (e) ssDNA1 (ssDNA). (f) DNA concentration-dependent A_695_ changes. [m-TPyEOPP] = 5 μM.

**3.3.2 Fluorescent titration spectra**

**Figure S19.** (a-e) DNA concentration-dependent fluorescence spectral changes of m-TPyEOPP (λ_ex_ = 454 nm) under the pH condition of 6.5. (a) Oxy28 (G-quadruplex); (b) C-MYC (G-quadruplex); (c) CtDNA (dsDNA); (d) dsDNA (dsDNA); (e) ssDNA1 (ssDNA). (f) DNA concentration-dependent F_720_/F_658_ changes. [m-TPyEOPP] = 5 μM.

**3.3.3 RLS titration spectra**

**Figure S20.** (a-e) DNA concentration-dependent RLS spectral changes of m-TPyEOPP under the pH condition of 6.5. (a) Oxy28 (G-quadruplex); (b) C-MYC (G-quadruplex); (c) CtDNA (dsDNA); (d) dsDNA (dsDNA); (e) ssDNA1 (ssDNA). (f) DNA concentration-dependent I_700_/I_650_ changes. [m-TPyEOPP] = 5 μM.

**3.4. FA-TMPipEOPP at pH 6.0**

**3.4.1 UV-vis titration spectra**

**Figure S21.** (a-e) DNA concentration-dependent absorption spectral changes of FA-TMPipEOPP under the pH condition of 6.0. (a) KRAS (G-quadruplex); (b) C-MYC (G-quadruplex); (c) CtDNA (dsDNA); (d) dsDNA (dsDNA); (e) ssDNA1 (ssDNA). (f) DNA concentration-dependent A_695_ changes. [FA-TMPipEOPP] = 5 μM.

**3.4.2 Fluorescent titration spectra**

**Figure S22.** (a-e) DNA concentration-dependent fluorescence spectral changes of FA-TMPipEOPP (λ_ex_ = 454 nm) under the pH condition of 6.0. (a) KRAS (G-quadruplex); (b) C-MYC (G-quadruplex); (c) CtDNA (dsDNA); (d) dsDNA (dsDNA); (e) ssDNA1 (ssDNA). (f) DNA concentration-dependent F_710_/F_658_ changes. [FA-TMPipEOPP] = 5 μM.

**3.4.3 RLS titration spectra**

**Figure S23.** (a-e) DNA concentration-dependent RLS spectral changes of FA-TMPipEOPP under the pH condition of 6.0. (a) KRAS (G-quadruplex); (b) C-MYC (G-quadruplex); (c) CtDNA (dsDNA); (d) dsDNA (dsDNA); (e) ssDNA1 (ssDNA). (f) DNA concentration-dependent I_700_/I_650_ changes. [FA-TMPipEOPP] = 5 μM.

1. **Aggregation behaviors of porphyrins in the presence of DNA**

**4.1. OMHEPzEOPP at pH 7.4**

**Figure S24.** RLS spectra of OMHEPzEOPP in the presence of different concentrations of individual DNAs. The inserts show the DNA concentration-dependent changes in the RLS signal intensity at 450 nm. [OMHEPzEOPP] = 5 μM.

- 1. **TMPipEOPP at pH 7.0**

**Figure S25.** RLS spectra of TMPipEOPP in the presence of different concentrations of individual DNAs. The inserts show the DNA concentration-dependent changes in the RLS signal intensity at 450 nm. [TMPipEOPP] = 5 μM.

**4.3. m-TPyEOPP at pH 6.5**

**Figure S26.** RLS spectra of m-TPyEOPP in the presence of different concentrations of individual DNAs. The inserts show the DNA concentration-dependent changes in the RLS signal intensity at 450 nm. [m-TPyEOPP] = 5 μM.

**4.4. FA-TMPipEOPP at pH 6.0.**

**Figure S27.** RLS spectra of FA-TMPipEOPP in the presence of different concentrations of individual DNAs. The inserts show the DNA concentration-dependent changes in the RLS signal intensity at 450 nm. [FA-TMPipEOPP] = 5 μM.

1. Circular dichroism (CD) analysis of the TMPipEOPP/KRAS mixture

The CD spectrum of KRAS showed a positive peak at 266 nm and negative peak at 243 nm, which is a typical characteristic of parallel G-quadruplex structure. KRAS kept this parallel G-quadruplex structure after addition of increasing concentrations of TMPipEOPP, which was reflected by the little effects of TMPipEOPP on KRAS CD spectrum (Figure S28a). Such a G-quadruplex structure was also irrelevant with KRAS concentration. As shown in Figure S28b, with the increase of KRAS concentration, the CD signal intensities of both positive peak and negative peak increased, but their wavelengths showed no changes.

**Figure S28.** CD spectra of the TMPipEOPP/KRAS mixture. (a) KRAS is kept at 5.0 μM and the TMPipEOPP concentration is varied from 0 to 10.0 μM. (b) TMPipEOPP is kept at 5.0 μM and the KRAS concentration is varied from 0 to 10.0 μM.

1. **Job Plot analysis for the binding interaction between OMHEPzEOPP or TMPipEOPP and KRAS**

**Figure S29.** Job Plot analysis for the binding interaction between OMHEPzEOPP or TMPipEOPP and KRAS.

1. **RLS titration spectra of other G-quadruplex probes**

**Figure S30.** RLS spectra of (a) thioflavin T (ThT), (b) crystal violet (CV) and (c) malachite green (MG) in the presence of different concentrations of KRAS. [ThT] = [CV] = [MG] = 5 μM.

1. **Improvement of RLS-based G-quadruplex-probing by addition of non-G-quadruplex DNAs**

**Figure S31.** KRAS-probing in the absence or presence of ssDNA1. (a) KRAS concentration-dependent RLS spectral changes of TMPipEOPP in the absence of ssDNA1. (b) KRAS concentration-dependent RLS spectral changes of TMPipEOPP in the presence of 5 μM ssDNA1. (c) KRAS concentration-dependent changes in RLS intensity ratio of I_702_/I_652_ in the absence or presence of ssDNA1. [TMPipEOPP] = 5 μM.

1. **Fluorescent and colorimetric G-quadruplex-probing in the presence of irrelevant DNAs**

**Figure S32.** Fluorescent and colorimetric sensing of KRAS in the presence of 5 μM ssDNA1. (a) KRAS concentration-dependent changes in the fluorescent spectrum of TMPipEOPP. (b) KRAS concentration-dependent changes in the fluorescent intensity ratio of F_702_/F_660_. (c) KRAS concentration-dependent changes in the absorption spectrum of TMPipEOPP. (d) KRAS concentration-dependent changes in the absorbance at 693 nm. [TMPipEOPP] = 5 μM.

1. **Let-7a-sensing using RLS probes**

**10.1. Oligonucleotides used in microRNA-sensing assay**

**Table S1.** The oligonucleotides used in Let-7a-sensing assay

| DNA or RNA | Sequence (from 5′ to 3′) | Extinction coefficient  [L·mol^-1^·cm^-1^] |
| --- | --- | --- |
| ON1 | AACTATACAACCTACTACCTCAAGGGCGGTGTGGGAAGAGGGAA | 446200 |
| ON2 | CCGCCCTTGAGGTAGTA | 161300 |
| Let-7a | TGA GGT AGT AGG TTG TAT AGT T | 226500 |
| Let-7g | TGA GGT AGT AGT TTG TAT AGT T | 224500 |
| Let-98 | TGA GGT AGT AAG TTG TAT TGT T | 222500 |
| Let-7i | TGA GGT AGT AGT TTG TGC TGT T | 212600 |

**10.2. Let-7a-sensing using OMHEPzEOPP as RLS probe**

**Figure S33.** Let-7a detection using OMHEPzEOPP as RLS probe. (a) RLS spectra of the sensing solutions in the presence of different concentrations of Let-7a. (b) Let-7a concentration-dependent changes in RLS intensity ratio of I_702_/I_658_. The insert shows the linear relationship between I_702_/I_658_ value and Let-7a concentration in the range of 0-3.5 μM. [OMHEPzEOPP] = 5 μM.

**10.3. Specificity of Let-7a assay**

**Figure S34.** Specificity of the proposed Let-7a-sensing platform. (a) RLS spectra and (b) I_702_/I_652_ values given by the sensing solutions in the presence of different microRNAs. Herein, TMPipEOPP was used as the RLS probe.
